# A Finite Element Model for Predicting the Static Strength of a Composite Hybrid Joint with Reinforcement Pins

**DOI:** 10.3390/ma16093297

**Published:** 2023-04-22

**Authors:** Francesco Bianchi, Yiding Liu, Adam M. Joesbury, David Ayre, Xiang Zhang

**Affiliations:** 1 Composites and Advanced Materials Centre, School of Aerospace, Transport and Manufacturing, Cranfield University, Bedford MK43 0AL, UK; 2 School of Physics, Engineering & Computer Science, University of Hertfordshire, Hatfield AL10 9AB, UK; 3 Centre for Manufacturing and Materials, Coventry University, Coventry CV1 5FB, UK

**Keywords:** composite hybrid joints, pin-reinforcement, static strength, FEA, experiment

## Abstract

This paper presents a finite element model for predicting the performance and failure behaviour of a hybrid joint assembling fibrous composites to a metal part with reinforcement micro pins for enhancing the damage tolerance performance. A unit-strip model using the cohesive elements at the bond interface is employed to simulate the onset and propagation of debonding cracks. Two different traction–separation laws for the interface cohesive elements are employed, representing the fracture toughness properties of the plain adhesive bond and a pin-reinforced interface, respectively. This approach can account for the large-scale crack-bridging effect of the pins. It avoids using concentrated pin forces in the numerical model, thus removing mesh-size dependency, and permitting more accurate and robust computational analysis. Lap joints reinforced with various pin arrays were tested under quasi-static load. Predicted load versus applied displacement relations are in good agreement with the test results, especially for the debonding onset and early stage of crack propagation.

## 1. Introduction

One of the major issues in using composite materials for aerostructures is their integration with metallic components. Conventional joining techniques such as adhesive bonding and mechanical fastening have major drawbacks. Adhesively bonded joints do not have sufficient strength at the joint runout regions due to the effect of stress concentration [1,2,3]. Inherent risk of delamination or debonding exists, together with poor damage tolerance capability [4,5]. On the other hand, mechanical fastening with bolts or metallic inserts adds weight; the hole drilling can cause damage to the laminate and raise local stress by a factor of three [6]. Strength reduction due to holes is much greater for laminated composites than metals due to composites’ being more sensitive to the notch effect [2]. 

Development of new joining methods is vital to promote applications of composite materials in aerospace and automotive structures. Hybrid joining techniques such as bonding plus bolting [7,8], interleaving [9,10,11,12] and surface structuring [6,13,14,15] have been developed, offering some compromise between reduced weight and increased structural redundancy. To avoid drilling fastener holes in composites, surface-structuring technology has attracted much attention. It is a combination of adhesive bonding and mechanical reinforcement by inserting small metal pins into the composite laminate to form an “interlocking system” to bridge debonding cracks, consequently increasing the resistance to fatigue crack growth rate. These through-thickness reinforcements with either metallic or fibre-polymer pins (referred to as micro pins) are typically 0.28–1.5 mm in diameter, to join two similar or hybrid materials together [16]. A variety of reinforcement pins have been developed over the past decades. The Surfi-Sculpt™ technique used protruded or sculpted surface features such as blunt pins made with a laser beam [13,14]. Pins can also be made by additive manufacturing [6,17], arc welding (cold metal transfer, CMT [15,18,19]), cold discharge stud weld (CDSW) [20] or sheet pins [21]. The hybrid penetrative reinforcement (HYPER) pin is one such technology that involves using small pins that are built onto a titanium substrate by additive manufacturing. HYPER pins only penetrate partway through the thickness of the laminate, providing an aerodynamic benefit compared to even a countersunk fastener. The sheet pins are produced by stamping a specific pattern on a metal sheet and then rotating the reinforcing elements perpendicular to the substrate, which has the advantage of having the reinforcement element unaffected by mechanical or thermal stresses during manufacturing [22]. 

**Figure 1:**
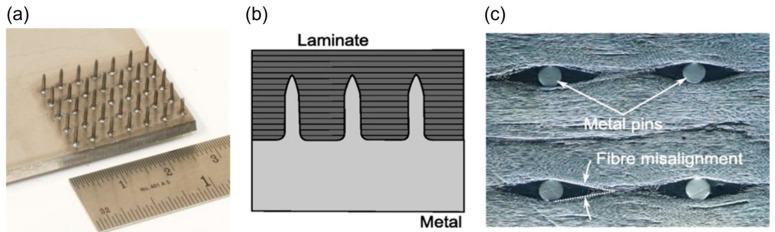
The concept of metal-composite joint reinforced with micro pins: (**a**) metal plate and reinforcement pins manufactured by the cold metal transfer (CMT) process [18], (**b**) schematic diagram of a pin-reinforced metal-composite joint, (**c**) resin-rich pockets and fibre movement due to inserted pins.

One of the promising manufacturing technologies to produce these small pins and integrate them as part of the metal part is a welding technique called cold metal transfer (CMT) [15,18]. Micro pins are welded onto the surface of a metallic part (Figure 1a) and then pushed into the composite laminate (Figure 1b) during joint assembly with a special ultrasonic gun before the joint is cured at an elevated temperature. The high-frequency vibration of the ultrasonic gun produces heat at the contact points between the pins and laminate. The local heat reduces the resin viscosity, allowing pins to be pushed in, causing minimal fibre breakage. The in-plane fibres must move around the inserted pins, resulting in small resin-rich pockets around the pins. The resin-rich zones have an elongated shape, extending in the local fibre direction (Figure 1c). 

The function of these metal pins is similar to that of the Z-Fiber^®^ pinning technology (also known as z-pins) used to reinforce composite laminates [5,23,24,25], in which the pins bridge the delamination cracks by exerting traction forces at the crack wake. The bridging forces shield the crack opening displacement and decrease the crack-tip strain energy release rate, hence increasing the joint strength and damage tolerance capability; the latter is a mandatory requirement for passenger aircraft structures. 

Cohesive zone modelling (CZM) is currently the most powerful technique for static fracture modelling of adhesive joints [26,27]. The cohesive laws take advantage of mixing a stress criterion for crack initiation and a fracture criterion for crack propagation. The main advantage of CZM is mesh independence, since damage growth is predicted by an energy-based criterion averaged over an area, instead of stress-based concepts [28]. In recent years, more complicated traction–separation curve shapes were proposed to increase model accuracy over the traditional CZM curves, such as the Park–Paulino–Roesler law [29], which includes a parameter to vary the softening curvature, thus enabling the simulation of varying adhesive plasticity. A review of various cohesive zone models and associated traction–separation curves for implementation in the cohesive elements can be found in [30], in which a new traction–separation law was also proposed that covers the linear and exponential softening models provided in the software library of ABAQUS v6.9. As the model contains a three-stage softening part, most of the experimental test results with linear, polynomial or exponential behaviour can be modelled. 

The key input into a cohesive zone model is the traction–separation law, expressed as stress vs. crack-opening displacement. The successful use of cohesive zone modelling relies on this traction–separation law to represent accurately the fracture of the material or interface. The traction–separation law therefore must be calibrated experimentally. Standard test methods for determining traction–separation curves use the double cantilever beam (DCB) specimen for mode-I, end notch flexure (ENF) for mode-II, and mixed-mode bending (MMB) for mixed modes I/II. These standard tests are used for testing the adhesive bond strength without reinforcement pins [31]. For pin-reinforced joints, single-pin pullout tests were undertaken to obtain the pin’s traction–separation laws in either mode-I or mode-II. To improve the computational efficiency and overcome the difficulties caused by mesh dependence, a moving mesh approach was proposed recently to model z-pin-reinforced specimens. It can also reduce the computational cost and potentially be implemented in realistic structures in pin reinforcement zones [32]. To incorporate small pins in structural models, single-pin bridging behaviour is characterised by the pin’s traction–separation curves. Nguyen et al. studied specimens containing a titanium pin in carbon-epoxy laminate by means of finite element modelling [33]. The model delivered traction–separation laws that are in good agreement with the experiments in mode-I and mode-II. 

Experimental studies on composite-to-metal joints reinforced with micro-sized pins (micro pins) have also been carried out recently. A novel joint of carbon-epoxy laminate and aluminium alloy was reinforced with an array of steel micro pins of 0.51 mm diameter inserted into pre-drilled holes to achieve an interference fit of 1%. Both the ultimate strength and ductility were increased under lap shear and peel load conditions [34]. In another study of carbon-fibre composite and aluminium joints, fatigue tests were performed in addition to static load tests; both tests demonstrated the beneficial effect of penetrative thin pins [35]. Another joining method used metal pins that were additively manufactured with different pin diameters and tip geometries, which were inserted into the locally infrared-heated composite part [17]. The study focused on the effect of fibre orientation on joint efficiency.

This paper presents a finite element model to simulate the enhanced damage tolerance capability and failure process of pin-reinforced metal-composite joints. The model presented in Section 3 employs the cohesive failure elements at the bondline interface. Two different cohesive failure laws are used: one represents an unreinforced adhesive bond, and the other models the pin-enhanced fracture toughness, which is measured experimentally to find the bridging law parameters of a single-pin configuration that quantitatively describes the pin’s energy absorption rate (enhanced toughness) during its failure process, from its debonding from the laminate to its being pulled out from the laminate. Section 4 shows the modelling results and parametric studies. Section 5 presents experimental investigation of the effect of pin array arrangement on the joint strength. Content in Section 2, Section 3 and Section 4 is an extension of a conference paper [36]. Work reported in Section 5 is new from a co-author’s PhD thesis that has not been published. 

## 2. Materials and Test Specimens

**Figure 2:**
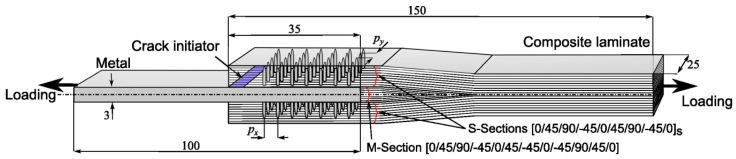
Schematic of composite-metal lap joint and dimensions; pin pitch distances (*p_x_* and *p_y_*) are variables; their values are given in the next section (unit: mm).

**Figure 3:**
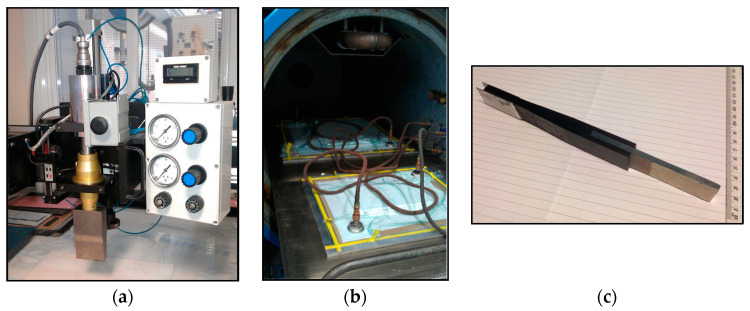
Test specimen manufacture using: (**a**) ultrasonic horn, (**b**) autoclave. (**c**) An example of specimen ready for mechanical testing.

Test specimens representing the pin-reinforced lap joint were fabricated and tested [18]. Figure 2 shows the specimen geometry and dimensions. An unreinforced joint was also tested as a baseline to evaluate the effect of the pins. The test method was informed by ASTM D5868, with adaptation of a larger interface area to accommodate pin array patterns. Carbon-fibre epoxy prepreg (Hexcel Corporation HexPly^®^ T700/M21, Hexcel, Duxford, UK) and steel alloy AISI 304 were used to fabricate the joints. The adhesive bond was strengthened with pins arranged in a 5 × 7 array. Spike-headed pins of 0.8 mm diameter and 4 mm height were welded onto the surface of the metallic part using the Fronius International GmbH CMT pin process [37]. The metal part of the overlap was sand-blasted to improve the adhesion between the composite and metal part. The composite part forms the middle section (M-section) and two side sections (S-section); it has quasi-isotropic layups: [0/45/90/−45/0/45/−45/0/−45/90/45/0] for the “M” and [0/45/90/−45/0/45/90/−45/0]_S_ for the “S” sections. Nominal ply thickness was 0.25 mm. An initial crack of 5 mm length was created by inserting a thin FEP (fluorinated ethylene propylene) film at the runout of the composite part, as indicated in Figure 2. A Branson ultrasonic horn with a ‘hammer’ attachment was used to insert the pins into the prepared laminate, which did not cause damage to the fibres (Figure 3a). The assembled specimens were then enclosed within a vacuum bag and cured in an autoclave, following the manufacturer’s specified cure schedule of 150 °C for 180 min, followed by 180 °C for 180 min, under an autoclave pressure of 7 bar throughout (Figure 3b). The prepreg epoxy matrix system was used as the joint adhesive, therefore forming a co-bonded joint simultaneously during the laminate cure cycle (Figure 3c).

All specimens were tested under static tension load using a 100 kN test machine under the displacement-controlled loading condition at loading speed 1 mm/min. Debond crack propagation was measured with an optical microscope. 

## 3. Model Descriptions

### 3.1. Single-Pin Model

**Figure 4:**
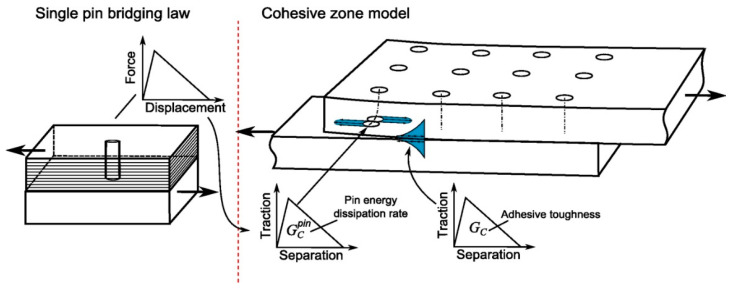
Multi-level modelling approach showing a single-pin model (**left**) and a joint model (**right**).

**Figure 5:**
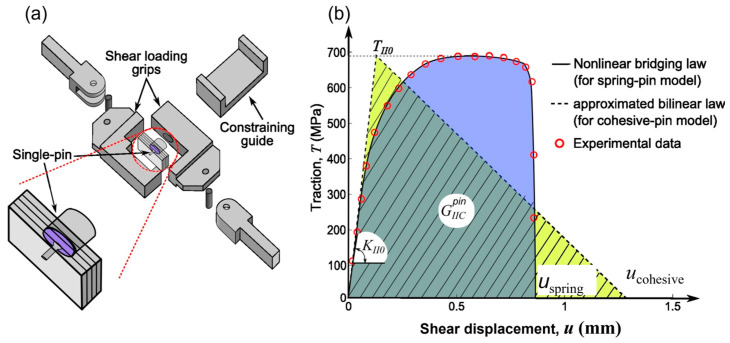
Single-pin bridging law test: (**a**) schematic of the testing rig, (**b**) pin bridging traction–separation laws used for the two different pin models (one is based on the spring pin behaviour, the other on the cohesive pin behaviour).

It is assumed that the crack-bridging force of the pins can be characterised by the load-carrying capability of a collection of single pins, as shown in Figure 4. The crack-bridging force exerted by a pin was measured by testing single-pin specimens under the mode-II loading condition [18]. The adhesive bond in the double-lap joint mainly suffers from the shear-driving fracture, i.e., the mode-II loading condition, especially at the early and middle stages of crack growth. The test rig is shown in Figure 5a. The specimen is a square block laminate (20 × 20 × 4.6 mm^3^) with the same layup as the composite part of the joint and contains a metal pin of the same material and same pin size as the pins used in the joint. The single pin was welded by the CMT process onto a thick cylindrical metal substrate (of 12 mm diameter and 12 mm height). This specimen can be regarded as a single pin unit cell. The test was carried out by constraining the crack opening displacement to avoid a mixed-mode load condition. Due to the yielding and bending deformation of the metal pin, the pin bridging force showed high nonlinearity in the rising part of the force–displacement relation (Figure 5b). 

To implement this nonlinear traction–separation law in the FE model of the joint, a governing equation in terms of pin stress versus crack displacement is deduced from the measured pin force:

(1) $$T(u)=\frac{P(u)}{\pi r_{0}^{2}}$$

where *u* is the shear displacement, *T*(*u*) the pin bridging stress and *P*(*u*) the bridging force. Equation (1) is used to represent a fitting curve to test data, as plotted in Figure 5b (solid line), based on the experimental test data shown by the symbols. The key parameters in the bridging law are the initial stiffness in the linear-elastic part and the yield load. Since the double-lap joint is mainly under the shear load (mode-II), the pin’s bridging law used in the models presented in this paper was based on the single-pin mode-II test [38]. Three experiments were performed in [38] and with a FE model in [39]. Considering the variability in the measured bridging curves in the nonlinear response part (i.e., large scatter for the ultimate load), only the linear-elastic part (the initial stiffness) and the yield load were considered an accurate representation of the pin’s bridging law, i.e., as the key parameters, which were used in the model in this paper. 

To implement the single-pin bridging law in the joint (or structural) models, two different models were used. The “spring pin” model represents the test-measured nonlinear bridging curve; the “cohesive pin” is the bilinear curve (dashed line), which approximates the nonlinear bridging law. Curves representing the two bridging laws are equivalent in terms of the pin-enhanced fracture toughness ($G_{IIC}^{pin}$), which is the area under the stress–displacement curve; both curves have the same energy absorption rate due to the pin bridging and pullout effect. 

### 3.2. Structural Model of Pin-Reinforced Joints

First of all, the unpinned joint was modelled by a plane-strain model. The model was used to calibrate the cohesive element properties of the adhesive bond and the cohesive failure model parameters according to the test result. Material properties of the adherends are given in Table 1. Plastic deformation of the metal part was modelled by an iterative procedure using the Ramberg–Osgood stress-strain relationship [40]:

(2) $$\varepsilon =\frac{\sigma }{E}+0.002{\left(\frac{\sigma }{\sigma_{Y}}\right)}^{n}$$

**Figure 6:**
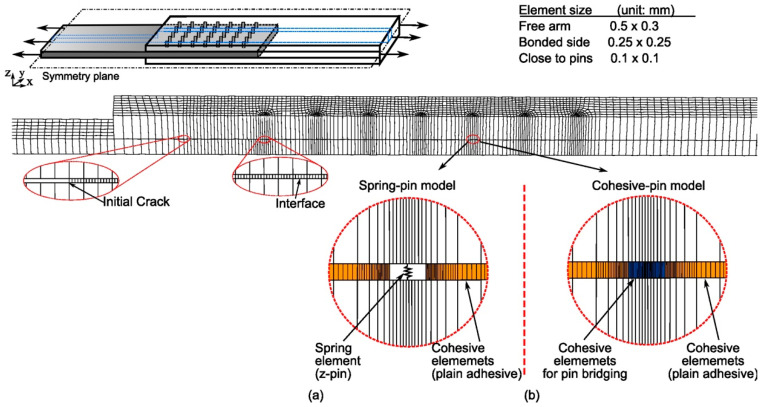
A unit-strip model of pin-reinforced composite-metal lap joint; inserts (**a**,**b**) show the two different single-pin models used in the joint model.

The model of the pin-reinforced joint has the same geometry as the unpinned joint, but the plane-strain model for the unpinned joint cannot be used because of the pins. According to the periodic placement of the pins, a unit-strip model is used to represent a “periodic unit” of the joint that contains half of a pin row. As shown in Figure 6, the unit-strip model consists of pins placed at a regular interval according to the pin pitch in the longitudinal direction (e.g., *p_x_* = 3.75 mm), and the width of the strip is half of the pin pitch in the joint lateral direction (*p_y_* = 4.16 mm). However, it should be noted that this model represents a pinned joint of infinite width (i.e., similar to a plane-strain model). Model details can be found in [36,39,41,42]. 

Eight-node linear continuum shell elements with reduced integration (designated as CS8R in ABAQUS) were used for the composite and metal adherends, whereas the adhesive bond interface was modelled by a layer of 8-node cohesive elements (COH8). The boundary condition of the unit-strip model was set according to the periodic pin arrangement to represent an infinitely wide joint. The nodal displacement at periodical boundaries is constrained in the *y*-direction. The two planes that delimit the unit-strip are constrained to remain in-plane, but also allowed to contract laterally due to the Poisson effect. Half of the specimen was modelled, owing to the symmetry; the vertical displacement of the symmetry plane is constrained. Numerical simulation was performed applying a monotonically increasing displacement at the joint ends.

The pin bridging law, Equation (1), was implemented in the global FE model of the joint specimen using two different single-pin models representing the nonlinear and bilinear bridging laws, as shown in Figure 6 inserts (a) and (b), respectively. The “spring pin” model employs nonlinear spring elements with a user-defined force–displacement relation representing the nonlinear curve in Figure 5b. As depicted in Figure 6a, these springs are connected to the two adherends through the multiple-point constraints. The “cohesive pin” model represented by a bilinear traction–separation law using cohesive interface elements, Figure 6b, is easier to implement in the commercial software package ABAQUS. The suitability of these two different single-pin models is discussed in Section 4. The plain adhesive was modelled by cohesive elements with the cohesive properties in Table 1. 

The reaction or applied force on the joint (*P*) is calculated by the unit-strip model (*P_strip_*) by Equation (3):

(3) $$P=\frac{W}{0.5p_{y}}P_{Strip}$$

where *W* is the joint width and *p_y_* the pin pitch in the width direction. The scaling factor is simply the ratio between the joint and the model width, i.e., the number of strips over the joint width.

Due to the finite width of the structure, the number of pins bridging the crack over the joint width (*N_pin_*) generally differs from the scaling factor calculated by Equation (3). To account for this influence, the pin bridging traction used in the unit-strip model (*T_strip_*) is corrected by Equation (4). It is worth noting that this factor can vary in the range between (*N_pin_* ± 1) and *N_pin_*. For wider joints, such as aircraft fuselage or wing panels, this correcting factor is close to unit.

(4) $$T_{strip}=\frac{0.5p_{y}}{W}N_{pin}T$$

## 4. Modelling Results

### 4.1. Force vs. Applied Displacement

**Figure 7:**
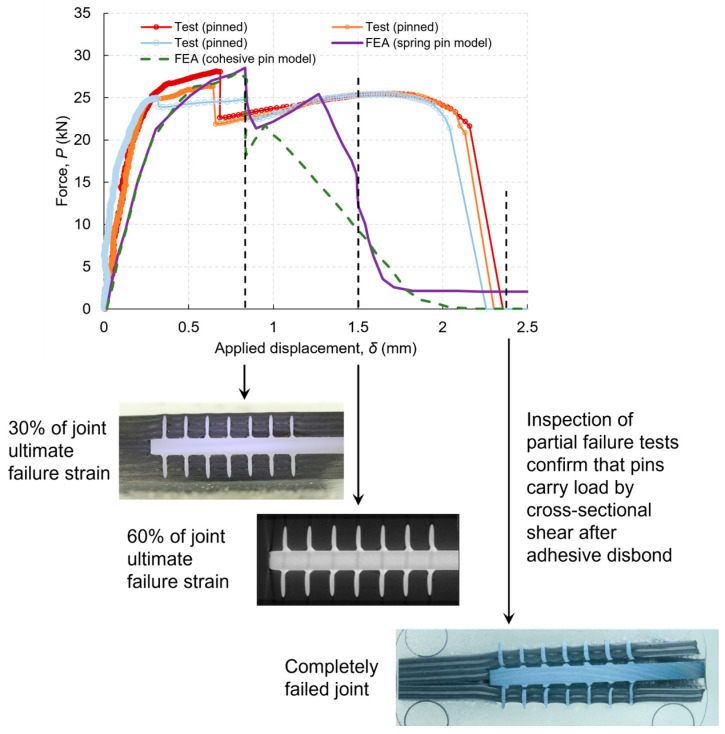
Predicted force vs. displacement relations and comparison with tests. Inserted images show the pins at various stages of the test, demonstrating that the pins carried the load after the initial debonding. (Metal adherend thickness 3 mm).

Figure 7 shows the reaction force vs. applied displacement relationship. First, the agreement between the predicted and experimental is very good in terms of the peak load points. Both the cohesive-pin model and spring-pin model predicted similar force–displacement responses up to the peak load point, with 5% difference between the prediction and test. The sudden load-drop immediately after the peak load corresponds to debonding onset. Second, both models predicted the load recovery process after the initial debond damage. From this point onwards, the load is completely carried by the pins’ bridging traction forces. The spring-pin model has better agreement with the test result, whereas the cohesive-pin model predicts a stiffer response post the peak load and a smaller displacement at final failure. This is owing to the cohesive-pin model’s employing a simplified bilinear traction–separation law. However, the comparison also shows that the models are less stiff than the test specimens, as shown in the initial rising part of the load vs. displacement curves before reaching the peak load point. This might be due to the elastic modulus in models being lower than the testing materials, including the adhesive and/or the material thickness variations. 

### 4.2. Interlaminar Shear Stresses

**Figure 8:**
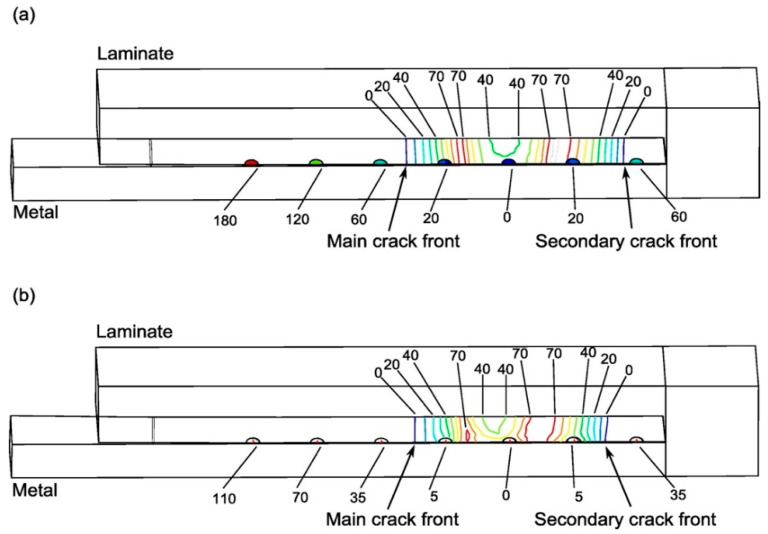
Shear stress distribution over the bond interface (contour map with stress values being indicated), and pin bridging stresses (marked under each pin); (**a**) cohesive-pin model, (**b**) spring-pin model (unit: MPa, main delamination crack is on the left, with length of 14 mm).

Figure 8 shows the interlaminar shear stresses at the bonding plane, with an applied displacement of 0.65 mm (main crack length 14 mm). Higher stresses were predicted by the cohesive-pin model on the pin rows close to the crack tip, owing to the bilinear cohesive-pin model being stiffer than the spring-pin model before the force reaches its peak (Figure 5b). It is worth noting that the pin stresses in Figure 8 should not be interpreted as the pin internal stress, but rather as an equivalent stress derived from dividing the bridging force by the pin cross-sectional area. The figure reveals the crack growth retardation mechanism of the pins’ traction forces in the crack wake (left side of the main crack front) picking up higher stresses. It also shows the failure behaviour of the joint, i.e., as the applied load increases, a secondary debond crack initiates at the other runout and propagates toward the main crack front. Final failure occurs when the two crack fronts meet at the centre of the joint. This behaviour is predicted by both the cohesive-pin and the spring-pin models.

### 4.3. Parametric Studies by FEA and Discussions

To better understand the pin bridging mechanisms and the way to improve joint performance, a parametric study was conducted by FEA. Bridging parameters are categorised as either geometrical or physical. The geometrical parameters are relative to the macroscopic dimensions, such as the adherend thickness, overlap length and pin pitch, whereas the physical parameters are associated with the pin’s traction–separation law: the initial stiffness ($K_{IIC}^{pin}$), cohesive strength ($T_{IIC}^{pin}$) and fracture toughness ($G_{IIC}^{pin}$). 

#### 4.3.1. Thickness of Metal Adherend

**Figure 9:**
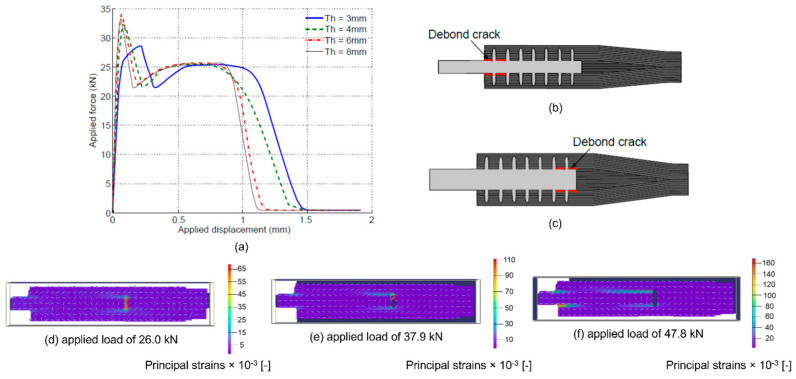
(**a**) Effect of metal thickness on the joint peak force, (**b**) crack initiation from the laminate runout with thin metal adherend, (**c**) crack initiation from the metal runout with thicker metal adherend. Strains in load direction measured by digital image correlation (DIC) for joints with 5 × 7 array micro pins with a 6 mm thick metal adherend under an applied load of (**d**) 26.0 kN, (**e**) 37.9 kN, and (**f**) 47.8 kN (metal adherend thickness 6 mm).

The FE analysis has shown that the onset of delamination occurred when the stress exceeded the yield strength of the material, which was manifested by large plastic deformation. The large deformation increased the local shear stress at the bond interface at the runout location. Joint response against metal thickness is shown in Figure 9a. Increasing the metal thickness makes the joint stiffer, and higher peak loads are achieved, whereas large plastic deformation in the thinner adherend showed a lower peak force. Beyond the peak load, the joint response is less sensitive to the metal thickness. This confirms that after the bondline failure (i.e., beyond the peak load), the strains are carried by the pins, and the joint response is totally characterised by the pin bridging force and the number of pins. 

Metal adherend thickness also influences the location of debonding onset; joints with thin adherend tend to debond from the laminate runout, as depicted in Figure 9b, whereas a thicker adherend promotes cracking from the opposite runout (Figure 9c). This behaviour is owing to the stiffness change; a higher shear stress peak corresponds to higher axial strain, whereas increasing the thickness reduces the peak stress at the laminate runout; hence, the debond crack starts from the opposite side. The analysis showed that a metal thickness of 6 mm achieved the maximum strength. Therefore, this thickness was used for all following analyses. Details can be found in [39]. 

Digital image correlation (DIC) was used to examine the side-on edge of a 5 × 7 pin array during mechanical testing. A Dantec Dynamics Q-400 DIC system was used to capture the series of images that were subsequently processed using ISTRA 4D software (Istra 4.2.3.82 Cranfield University). It can be seen from the processed images of Figure 9d–f that the debond initiates and propagates from the metal runout end of the joint, confirming the FE prediction for debond behaviour when a 6 mm adherent is used. Details can be found in [18].

#### 4.3.2. Pin Row Number

**Figure 10:**
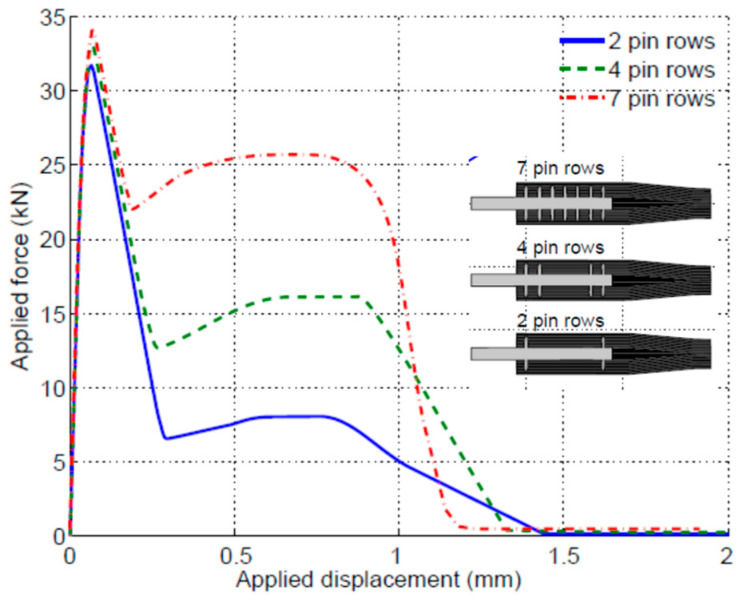
Effect of pin row numbers on the performance of the joints (metal adherend thickness 3 mm).

The experiments and FE analysis have demonstrated that at the bondline failure, no pin was completely pulled out (Figure 7), and stresses in the pins were still within the elastic limit. The most stressed pins were the ones near the joint runouts, where the shear deformation was the largest. The purpose of this modelling work was to understand the relative importance of the pin row numbers and locations in terms of their effectiveness in bridging debond cracks. Figure 10 shows the influence of the pin row numbers and locations on the joint strength (peak load at which complete debond occurs) and load recovery after the peak load. The joint strength increases with the number of pin rows; however, the enhancement on the peak load is not pronounced, the increment per pin row is not proportional, and it decreases with the pin row number. The smaller benefit of more pin rows may be due to the larger distance from the runout: the further the pin is from the joint runout, the less bridging action it exerts. Load recovery after the bond failure is totally due to the pins’ load-carrying capability; for this reason, the distance from runout is irrelevant: the load recovery is proportional to the number of pins only. Details can be found in [39]. 

#### 4.3.3. Parameters of Single-Pin Traction–Separation Laws

As described before, the pin traction–separation laws can be characterised by three parameters: the initial stiffness ($K_{IIC}^{pin}$), the cohesive strength ($T_{IIC}^{pin}$) and fracture toughness ($G_{IIC}^{pin}$). A sensitivity study of these parameters was performed by FEA to understand how the variation in a single-pin parameter affects the response of a joint. 

**Figure 11:**
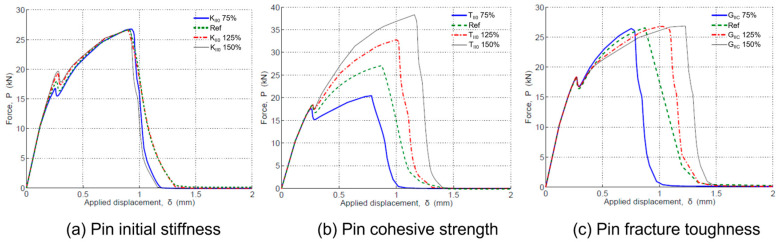
Joint performance against variations of single-pin cohesive law parameters: (**a**) initial stiffness, (**b**) cohesive strength, (**c**) fracture toughness.

Variation in the initial stiffness is found to have negligible effect on the joint performance; only marginal difference is shown in the first load drop (see Figure 11a). The pin’s cohesive strength has strong influence on the joint response after the first load drop, which corresponds to the debonding damage (Figure 11b). Pin toughness does not affect the load vs. displacement relation of joints before the first load drop; after that point, a higher pin toughness results in higher displacement of the joint at failure. However, the joint ultimate strength (the failure load) is insensitive to the variations of mode-II fracture toughness of a single pin (Figure 11c). Details can be found in [39]. It should be noted that the delamination behaviour in a double-lap joint is mode-II dominating. However, when a delamination crack is longer, mode-I will play a role owing to the beam deflection. This is a limitation of this study.

### 4.4. Summary of Important Points Based on the Modelling Work

One of the advantages of this modelling technique is that the single-pin and structural joint performance are modelled separately, meaning that the model can be readily employed for structures of different sizes and parameters (e.g., pin diameter, areal density, pin row number, and materials) by implementing a single-pin traction–separation law in a global FE model of the structure. This modelling technique has been previously used for predicting delamination retardation of z-fibre-reinforced composites and achieved good prediction results [6,18,36,43]. 

**Figure 12:**
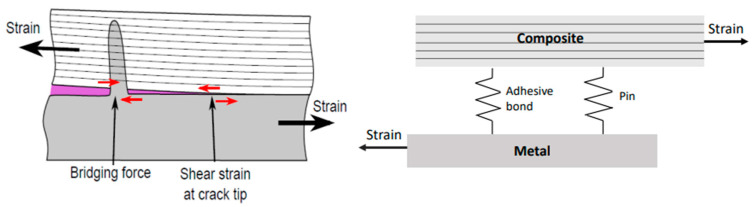
Schematic illustration of the effect of adherend axial stiffness on the effectiveness of pin traction in shielding the crack tip (**left**); the red arrows indicate the shear stresses. Insert figure (**right**) shows the two stress transfer elements: the pins and adhesive bond act like two springs working in parallel, transferring stress between the two adherends.

The model has shown that avoiding yield deformation of the metal adherend will increase the stiffness of the joint system, as depicted in Figure 12. This implies that at a fixed bridging force exerted by a pin (i.e., under fixed shear displacement), the shear strain at the crack tip is smaller if the joint system is stiffer. Therefore, increasing the axial stiffness of adherends, or yield strength of the metal part, makes the pins work better. The combined effect of the adhesive bond and pins may be seen as an analogy of two springs working in parallel (Figure 12 insert): one representing the pins, and the other the adhesive bond. If one is much stiffer than the other, then the two act at different stages (i.e., the one with lower stiffness fails much earlier), and there is no synergy between them, hence less benefit to the joint strength enhancement. On the other hand, if their stiffness values are similar, their synergistic action will increase the joint strength.

## 5. Experimental Observations on the Effect of Pin Array Arrangement

**Figure 13:**
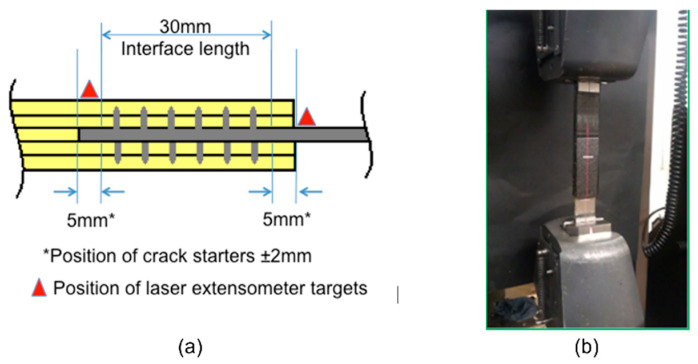
(**a**) Diagram showing details of the cross-section of the modified joint interface along the length of tensile adhesive joint test specimen, with the positions of the laser extensometer reflective targets; (**b**) the arrangement of the test setup.

It should be noted that the experimental results discussed in this section were based on a modified joint interface length of 30 mm, controlled by the usage of PTFE release tape at both ends of the joint overlap length to reduce the high local stress compared to the samples shown in Figure 2. The diagram with the details of the joint interface, as well as test setup, are shown in Figure 13. 

The parametric study here was used to design a series of structural joint configurations that would test the influence of micro-pin quantity and arrangement pattern on the joining performance. The metal thickness was chosen to be in line with the parametric study’s identified optimum of 6 mm. The geometry of individual micro pins was the same as previously used (in Section 2, Section 3 and Section 4), as was the hybrid-joint manufacturing method. The materials used were also the same specification. The differences for this series of tests were that the prepreg used was from a fresh batch that had less accumulated life, and the joints were constructed to have crack-initiating film placed at both the metal and the composite runout ends of the joint. 

**Figure 14:**
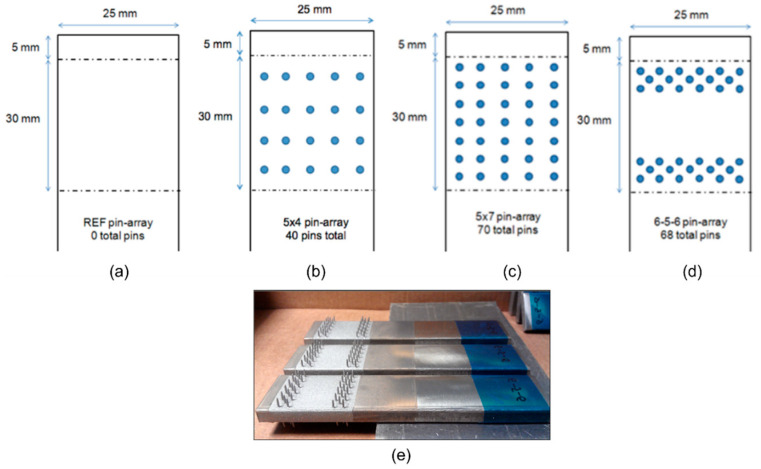
Schematics of micro-pin array patterns used in the experimental study: (**a**) no pins (Ref.), (**b**) 5 × 4 pin array, (**c**) 5 × 7 pin array, (**d**) 6-5-6 quincunx pin array, (**e**) the metal parts prior to joining with laminate in experiment.

Micro-pin arrays included in this test series were as follows: a reference sample with no pins (Figure 14a); four rows of five pins with regular spacing (total 40 pins; pins on both sides of the metal adherend), Figure 14b; seven rows of five pins with regular spacing (total 70 pins), Figure 14c; and a 6-5-6 quincunx pin array located at the bondline edges (total 68 pins), Figure 14d. The same testing methodology was employed as described in Section 2; the applied displacement was measured using a laser extensometer with reflective targets positioned to limit the measurement gauge to that of the joint overlap length. Details can be found in [18].

**Figure 15:**
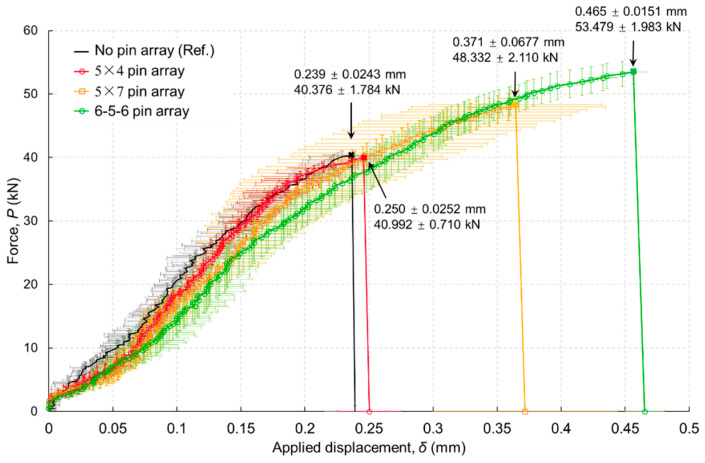
Load vs. applied displacement showing the effects of pin array: no pin array (Ref.), 5 × 4 pin array, 5 × 7 pin array, 6-5-6 pin array. Metal adherend thickness 6 mm.

Results of the mechanical testing, presented in Figure 15, showed that when relatively fewer pins are used, there is negligible effect on the joint strength (comparing the “no pin” to the “40 pins”). Joint strength is increased by use of a greater number of pins, and when the location of pins is concentrated at the bondline ends, the strength is further increased. 

The main difference in the load vs. displacement response between this test series with 6 mm thick metal adherend and the test with 3 mm thick metal adherend is the sudden load drop seen in the latter case (Figure 7) that corresponds to bondline failure; from that point, the pins take load to bridge the crack. The continuing load increase in Figure 15 can be explained as follows. First, the parametric study of the *single-pin* bridging law shows higher pin strength resulting in higher load capacity and the absence of sudden load drop that is seen at a lower pin strength (Figure 11b). The same effect can be said for the *joints* in Figure 15, where a large number of pins means increased pin strength. Second, the thicker metal adherend allows a more uniform shear stress distribution on the adhesive bond, and owing to the higher stiffness of the metal part (doubling the thickness), the joint system engages more pins to delay the onset of debonding as well as bridge the crack propagation after bondline failure. In summary, the difference between pins bridging crack propagation (thin metal adherend) and pins retarding crack onset (thick metal adherend) is owing to the synergy of the adhesive strength, metal substrate stiffness and engagement of the pins. 

The hybrid joint should be designed with a higher number of pins at the joint runout, where the shear stress is the highest and hence is the most critical for debonding damage. 

## 6. Conclusions

A finite element model has been developed and validated by an experimental test for a composite-metal lap joint with through-thickness reinforcement by micro pins. The developed modelling approach can be used to evaluate the design parameters of pin-reinforced composite-metal joints, specifically the substrate size and pin arrays. Additional experiments are also presented on the effect of pin array arrangement. Based on these studies, the following conclusions can be drawn:

1. The bridging force exerted by micro pins can be modelled by the nonlinear spring elements and the cohesive interface elements. Bridging laws governing these pin models can be obtained either by testing single-pin specimens or using the unit-cell models.
2. Both pin models can be implemented in a commercial finite element software package for modelling structural failure behaviour of lap joints.
3. The spring-pin model can better simulate the joint response to the complete failure of the joint, whereas the cohesive-pin model has less good agreement with the experimental curve, owing to the over-simplified traction–separation law for the single-pin model.
4. Increase in the pin numbers along the path of the debonding crack will increase the structural performance of the joint. The hybrid joint should be designed with a higher number of pins at the joint runout, where the shear stress is the highest, hence being the most critical for debonding damage.
5. A synergistic effect of the metal substrate stiffness and pin array variation on the joint strength and damage tolerance properties has been demonstrated by testing two different metal adherend thicknesses. This should be further investigated by modelling and parametric studies to optimise the design of hybrid joints.

## Figures and Tables

**Table 1:** Material properties and cohesive element parameters used in the FE model.

Property	Value
**Composite adherend** (unidirectional carbon fibre/epoxy Hexcel T700/M21)	
Young’s modulus in longitudinal direction (*E*_11_)	120 GPa
Young’s moduli in transverse directions (*E*_22_, *E*_33_)	11 GPa
Shear moduli (*G*_12_, *G*_13_, *G*_23_)	4.6 GPa
Poisson’s ratio (υ_12_, υ_13_, υ_23_)	0.35
**Metal adherend** (Stainless steel AISI-304)	
Young’s modulus (*E*)	190 GPa
Poisson’s ratio (υ)	0.33
Yield strength (*σ_Y_*)	290 MPa
Ramberg–Osgood parameter used in Equation (1) (*n*)	3.8
**Adhesive: cohesive element properties** (same material as the prepreg resin M21)	
Mode-I traction stiffness (*K_I_*)	2.5 × 10^13^ N/m^3^
Mode-II traction stiffness (*K_II_*)	2.5 × 10^13^ N/m^3^
Mode-I cohesive strength (*T_I_*_0_)	30 MPa
Mode-II cohesive strength (*T_II_*_0_)	70 MPa
Mode-I fracture toughness (*G_IC_*)	200 J/m^2^
Mode-II fracture toughness (*G_IIC_*)	550 J/m^2^

## Data Availability

Data will be provided based on request.
